# A model of the natural history of screen-detected prostate cancer

**DOI:** 10.1038/sj.bjc.6603368

**Published:** 2006-09-26

**Authors:** C Metcalfe, J A Lane, F Hamdy, D Neal, J L Donovan

**Affiliations:** 1 Department of Social Medicine, Bristol University, Canynge Hall, Whiteladies Road, Bristol BS8 2PR, UK; 2 Academic Urology Unit, Division of Clinical Sciences (South), University of Sheffield, K Floor, Royal Hallamshire Hospital, Glossop Road, Sheffield S10 2JF, UK; 3 Department of Oncology, Box 193, Addenbrookes Hospital, Hills Road, Cambridge CB2 2QQ, UK

**Sir**,

In their recent *British Journal of Cancer* paper, Parker *et al* (2006) were careful not to overplay the conclusions that can be drawn from their model of the natural history of screen-detected prostate cancer. They alerted the reader to the dangers of trying to learn about the effects of treatment on localised prostate cancer detected through prostate-specific antigen (PSA) testing when the results of randomised controlled trials are not available and data from observational studies are only available on men whose cancers were detected at a later stage of the disease. Parker *et al* also emphasised the importance of ongoing randomised trials, such as ProtecT (Donovan *et al*, 2003), which will provide direct and robust evidence of the effectiveness of different treatment approaches in men with PSA-detected disease. Regrettably, these notes of caution were absent from the ensuing coverage of the study in the UK media (Press Association, 2006).

In the large majority of cases detected with PSA testing, prostate cancer progresses very slowly, with death due to other causes most commonly intervening before the disease becomes life-threatening (Albertsen *et al*, 2005). PSA testing allows prostate cancer to be detected earlier, before symptoms develop, but there has been insufficient time since the introduction of testing in the 1990s for the most beneficial treatment approaches to be established through empirical studies. In this absence of observed data on the effects of treatment on PSA-detected disease, Parker *et al* (2006) utilised data on the 15-year survival of patients diagnosed at a later stage of their disease and managed conservatively (Albertsen *et al*, 1998), and data on the effect of radical *vs* conservative treatment on survival in men detected clinically (Bill-Axelson *et al*, 2005). These data informed a model that was used to predict the 15-year survival and effects of conservative and radical treatments in PSA-detected localised prostate cancer. Looking at survival and treatment effects in subgroups of men defined by their Gleason grade at diagnosis, Parker *et al* predicted that only 1% of those with lower grade (Gleason score less than 7) cancer would die of prostate cancer within 15 years, and concluded that there is no scope for these men to benefit from radical treatment.

A key feature of Parker *et al*'s (2006) model was the adjustment for the ‘lead time’ that PSA testing provides, that is, the amount of time by which PSA testing brings the diagnosis of prostate cancer forward, hence allowing the disease to be treated in its early stages. However, the estimated lead times may be too large, as they are based on all men in a cohort with localised prostate cancer irrespective of Gleason grade (Draisma *et al*, 2003). A proportion of the 13-year lead time observed in that cohort will be due to some cancers being diagnosed earlier *and at a lower grade* with PSA testing (Draisma *et al*, in press). Parker *et al*'s (2006) model incorporates Gleason grade at diagnosis, and so will capture that part of the effect of screening and, equivalently, lead time as more men diagnosed with lower grade cancer and so subject to the lower 15-year mortality rate observed for men of that cancer grade (Albertsen *et al*, 1998). Parker *et al* (2006) appear not to take this part-accommodation of lead time into account, using the full 13-year estimate from Draisma *et al*'s study (2003) to calculate category-specific lead times for men with different cancer grades at diagnosis. This ‘double-counting’ gives, for example, an estimated lead time of 14.1 years in men aged 55–59 years with lower grade cancer, with the model consequently only allowing death due to prostate cancer in the final 0.9 years of the 15-year observation period. This reduces the 15-year mortality from prostate cancer in that group of men from the 12% observed in the early study of conservative treatment (Albertsen *et al*, 1998) to a predicted 1% for contemporary cohorts with PSA-detected disease.

All models are simplifications of reality, but may still be useful despite their imperfections in the absence of trial evidence. It is this very lack of evidence, together with other less formal consideration of observational studies of conservative treatment strategies (e.g. Chodak *et al*, 1994), that convinced the ProtecT researchers to compare the effectiveness of active monitoring (i.e. no immediate intervention, with PSA monitoring that can trigger other treatments if the disease appears to be progressing) to that of established radical treatments, that is, prostatectomy and radiotherapy. Earlier observational studies created enough *doubt* in our current knowledge of the relative risk-benefit of immediate radical treatment for the ProtecT researchers to initiate a trial comparing conservative and radical approaches to treatment. This is in contrast to the press coverage resulting from Parker *et al*'s (2006) study that implied similar data *established* the relative benefits to be gained from the different treatments, without the need for randomised controlled trials. In particular, they question the suitability of the large majority of men with Gleason scores of less than 7 for radical treatment.

The ProtecT trial (comparing radical surgery, radical conformal radiotherapy and active monitoring) is still open to recruitment in the UK, as is Parker *et al*'s study of ‘active surveillance’ (Parker, 2004; Hardie *et al*, 2005). Men who read the press reports arising from Parker *et al*'s (2006) paper are likely to be misinformed about the strength of available evidence, and may wrongly assume that taking part in the ProtecT trial is unnecessary or even inappropriate. It remains the case that it is only through the conduct of a randomised study such as the ProtecT trial that we will be able to provide robust and directly relevant data to inform the management of contemporary cohorts of men with PSA-detected localised prostate cancer.
